# Sensory Processing and Aggressive Behavior in Adults with Autism Spectrum Disorder

**DOI:** 10.3390/brainsci11010095

**Published:** 2021-01-14

**Authors:** Frank van den Boogert, Bram Sizoo, Pascalle Spaan, Sharon Tolstra, Yvonne H. A. Bouman, Witte J. G. Hoogendijk, Sabine J. Roza

**Affiliations:** 1 Department of Psychiatry, Erasmus University Medical Center, 3015 GD Rotterdam, The Netherlands; f.vandenboogert@erasmusmc.nl (F.v.d.B.); p.spaan@erasmusmc.nl (P.S.); w.hoogendijk@erasmusmc.nl (W.J.G.H.); 2 Department of Research, Transfore, 7416 SB Deventer, The Netherlands; s.tolstra@transfore.nl (S.T.); y.bouman@transfore.nl (Y.H.A.B.); 3 Center for Developmental Disorders, Dimence Institute for Mental Health, 7416 SB Deventer, The Netherlands; b.sizoo@dimence.nl; 4 Netherlands Institute for Forensic Psychiatry and Psychology, 3511 EW Utrecht, The Netherlands

**Keywords:** sensory processing, sensory profile, aggression, aggressive behavior, autism spectrum disorder

## Abstract

Autism spectrum disorder (ASD) may be accompanied by aggressive behavior and is associated with sensory processing difficulties. The present study aims to investigate the direct association between sensory processing and aggressive behavior in adults with ASD. A total of 101 Dutch adult participants with ASD, treated in outpatient or inpatient facilities, completed the Adolescent/Adult Sensory Profile (AASP), the Reactive-Proactive Aggression Questionnaire (RPQ), and the Aggression Questionnaire—Short Form (AQ-SF). Results revealed that sensory processing difficulties are associated with more aggressive behavior ($f^{2}=0.25$), more proactive ($f^{2}=0.19$) and reactive aggression ($f^{2}=0.27$), more physical ($f^{2}=0.08$) and verbal aggression ($f^{2}=0.13$), and more anger ($f^{2}=0.20$) and hostility ($f^{2}=0.12$). Evidence was found for an interaction of the neurological threshold and behavioral response on total aggression and hostility. Participants with higher scores in comparison to the norm group in sensory sensitivity had the highest risk of aggressive behavior. In conclusion, clinical practice may benefit from applying detailed diagnostics on sensory processing difficulties when treating aggressive behavior in adults with ASD.

## 1. Introduction

Adults with autism spectrum disorder (ASD) are often confronted with problems in social, professional, and educational functioning and in physical and mental health [1]. In children with ASD, aggression is associated with more negative outcomes, such as decreased quality of life or less educational and social support [2]. More than half of all children with ASD demonstrate aggressive behavior directed at a caregiver or physical aggression in various situations [3,4]. When compared with typically developing children, aggressive behavior seems more common in children with ASD [5]. Compared with children with other intellectual and neurodevelopmental disorders, children with ASD showed more physical and reactive aggression [6]. In adults with ASD, there is no clear evidence for increased risk for aggressive or violent behavior [7]. However, on the level of an individual person with ASD, violent behavior could result from (undetected or untreated) third variables, e.g., family environment, criminality, psychiatric comorbidity [8], or various factors associated with ASD, such as younger age, repetitive behaviors, or sensory difficulties [7]. Research on factors that are associated with aggressive behavior in individuals with ASD might help inform treatment strategies, as too little is known about the underlying constructs or mechanisms to understand the association between ASD and aggression.

Specific phenotypic features, such as difficulties in sensory processing, may play an important role in the manifestation of aggression. Sensory processing difficulties are strongly associated with ASD and, from a more clinical point of view, considered to be part of ASD symptomatology. Sensory difficulties have therefore been added to the diagnostic criteria for ASD in the fifth edition of the *Diagnostic and Statistical Manual of Mental Disorders* (*DSM–5*) [9]. In general, sensory processing in persons with ASD differs from that in persons in the general population [10]. Sensory difficulties are present in the vast majority of children with ASD [11], and differences in comparison with children without ASD are seen on the full range of sensory processing issues [12]. Differences are largest for sensory under-responsivity, followed by sensory over-responsivity and sensory seeking [13]. Sensory difficulties in patients with ASD seem to persist through lifetime [11]. In adults with ASD, sensory differences were present in 94 percent of the population, although there is considerable diversity across individuals with ASD [14]. Sensory over-responsivity is particularly more common in adults with ASD in comparison to adults without ASD, and the severity of sensory over-responsivity is positively correlated with the level of autistic symptoms [15].

Sensory difficulties are a plausible, relevant phenomenon in the context of aggressive or violent behavior in persons with ASD. Mazurek, Kanne, and Wodka [4] found first indications of a positive association between sensory processing issues and aggressive behavior in children and adolescents with ASD. Gonthier et al. [16] reported a small positive correlation between sensation-seeking behavior and aggression directed towards others in a sample of adults with ASD and comorbid profound to severe intellectual disability.

Several models of sensory processing have been developed. Dunn’s Model of Sensory Processing [17,18] is among the most recognized models on this subject. In short, the model combines two continua: the vertical neurological threshold continuum for noticing of or reacting to stimuli, ranging from a low threshold or sensitization to a high threshold or habituation, and the horizontal behavioral response continuum, indicating the response to the neurological thresholds, ranging from responses in accordance with thresholds to responses to counteract the thresholds. The interaction of these continua results in a quadrant matrix: low registration, sensory seeking, sensory sensitivity, and sensory avoiding [18]. As a result, sensory processing issues are conceptualized, and measured, in several separate but interdependent factors. 

Although aggressive behavior is often analyzed on an aggregated level, aggression is a broad concept that allows specification and differentiation in various ways. An often-used differentiation discriminates between reactive and proactive aggression. Reactive aggression is an angry, impulsive, and defensive reaction to provocation, without thought of personal gain [19]. It is a response to poor emotion regulation, reduced self-control, diffuse sensory awareness, and heightened impulsivity [20]. Proactive aggression refers to instrumental, organized, and “cold-blooded” aggression, which is controlled by external reinforcements and is mostly not anger driven [21]. In the context of sensory processing, one might hypothesize that higher levels of sensory seeking behavior are associated with proactive aggression, whereas higher levels of sensory sensitivity or sensory avoiding behavior are associated with reactive aggression. When analyzing aggressive behavior in persons with ASD, we therefore distinguish between reactive and proactive aggression in the context of sensory processing issues. Our study aims to investigate the association between sensory processing difficulties and aggressive behavior in adults with autism spectrum disorder, using differentiated measurements of both sensory processing and aggressive behavior. To the best of our knowledge, this is the first study in which this association is investigated while applying important differentiations in the measurement of both concepts. Clinical practice may benefit from the acquired knowledge on the association between sensory processing difficulties and aggressive behavior in adults with ASD.

## 2. Materials and Methods

### 2.1. Procedure

The Sensory Processing and Aggressive Behavior in Autism Spectrum Disorders (SPAA) study is an observational study in a clinical population. Data were collected between April 2018 and April 2019 in outpatient and inpatient populations at units specializing in neurodevelopmental disorders at the Dimence Mental Health Care Institution in the Netherlands. Dimence is a general provider of mental health care for individuals with normal to high IQ (>70). At Dimence, specialized units provide assessment and treatment for adults with ASD. In general, inpatients are characterized by a lower level of functioning compared to outpatients.

Adult patients in treatment at the outpatient or inpatient facilities of Dimence for clinically diagnosed ASD who were willing to provide informed consent were eligible for participation in the study. The local protocol for the assessment of ASD in adults follows the national guidelines for ASD in adults [22]. The assessment is based on extensive diagnostic interviews by experienced clinicians. These interviews consist of a clinical interview with the individual, a detailed developmental history with a parent or other informant, and an interview on current functioning with someone who is well acquainted with the individual. Semistructured clinical interviews based on the Autism Diagnostic Interview—Revised (ADI-R) [23], a *DSM–5* checklist, and all available information from schools and child psychiatric services concerning childhood development are all important parts of the clinical procedure. The predefined exclusion criteria were insufficient knowledge of the Dutch language or other incapacities (e.g., due to psychosis, drug or alcohol intoxication, or intellectual disability) to understand the provided information. 

All therapists at the institution were extensively informed about the study and requested to select potential participants from their individual caseloads by applying the inclusion and exclusion criteria. Next, all selected patients were provided with the study’s information sheet by their own therapist. After sufficient reflection time, the patients were asked to consider participating in the study and, if they agreed, to fill out the informed consent form. Patients who provided informed consent were contacted by a research employee to schedule an appointment for completing the survey. During the appointment, a research employee was available at all times to provide brief verbal instructions and to answer questions. All participants were able to complete the survey. Participation was on a voluntary basis, and the participants received no benefit or compensation.

### 2.2. Participants

A total of 101 adult patients with a clinical diagnosis of ASD ($M_{Age}=32.9,\;SD_{Age}=12.4;\;N_{Male}/N_{Female}=53/48$) were included in the study sample. General sample characteristics are presented in Table 1. The percentage of participating females in our sample (48%) may reflect the increased attention for ASD in females, as well as the focus of Dimence on ASD patients with normal to high IQ [24]. Females were on average 5.3 years younger than males. The participants admitted to specialized psychiatric hospital units for the treatment of ASD (i.e., inpatients) were on average 5.4 years younger than the participants treated in outpatient facilities, were on average lower educated, and had more often no partner or spouse, and none of them had children. The groups defined by gender and by treatment setting, as presented in Table 1, did not differ with regard to comorbidity. Gender and treatment setting were not associated.

### 2.3. Measurements

We used the Dutch version of the Adolescent/Adult Sensory Profile (AASP) [25,26], a 60-item, self-report questionnaire to obtain information on responsiveness to various sensory stimuli and to identify difficulties in the sensory systems that may hinder an individual in daily functioning. The AASP is the most frequently used instrument model for this purpose in adults and adolescents with ASD [27]. The questionnaire produces four continuous subscale scores ranging from 15 to 75, representing the four quadrants of the Model of Sensory Processing [17,18]: low registration, sensory seeking, sensory sensitivity, and sensory avoiding. Each subscale consists of 15 items, rated on a 5-point Likert scale from never (1) to always (5). The values of the alpha coefficients for the quadrant scores range from 0.64 to 0.78 [28], which indicates satisfactory internal consistency.

Although the AASP is based on Dunn’s Model of Sensory Processing [17,18], the model’s two fundamental continua, the neurological threshold and behavioral response continua, are not measured directly but are represented in dichotomized form in the four continuous quadrant scores. For instance, the sensory seeking score consists of items measuring active behavioral responses in situations of lower neurological thresholds. Thus, whereas the model of sensory processing incorporates two dimensions resulting in four categories, the AASP measures these four categories as separate dimensions and, by doing so, transforms the original two dimensions from its underlying model into categories. This introduces several theoretical and methodological problems. In previous research, the quadrant scores were often analyzed separately, without taking the other quadrant scores into account from a theoretical or statistical point of view. However, as all four quadrant scores are based on the same underlying constructs and are therefore theoretically closely related, the quadrant scores would better be interpreted in conjunction with the other three scores. Additionally, application of the original continua would theoretically enable allocation of each individual to one of the quadrants to “type” the most prominent individual’s sensory processing pattern.

In line with previous research [29], we calculated the neurological threshold and behavioral response continua, each ranging from −120 to 120. Neurological threshold scores were calculated by subtracting the sum of the low neurological threshold quadrant scores from the sum of the high neurological threshold quadrant scores: (low registration + sensory seeking) − (sensory sensitivity + sensory avoiding). To calculate the behavioral response, we subtracted the sum of the passive behavioral response quadrant scores from the sum of the active behavioral response quadrant scores: (sensory seeking + sensory avoiding) − (low registration + sensory sensitivity). Raw quadrant scores were compared to age-related norm groups provided by the instrument’s manual and classified as much less than, less than, similar to, more than, or much more than the mean norm score [25]. According to the data published in the AASP manual, these classifications are based on the norm group mean scores and standard deviations. Likewise, the raw quadrant scores were converted into quadrant norm scores, ranging from −2 to +2. An aggregated-level variable for sensory processing difficulties, the sensory deviation score, was calculated by summing up all four quadrants’ norm score deviations from zero, in the range of 0 to 8. Finally, we calculated an AASP total score, in the range of 60 to 300, by summing up all raw quadrant scores.

The Reactive-Proactive Aggression Questionnaire (RPQ) [21] is a 23-item, self-report questionnaire for reactive and proactive aggression. All items were measured on a 3-point scale: never (0), sometimes (1), and often (2). The Dutch version of the questionnaire was used [30]. The instrument contains two subscales: proactive aggression, containing 12 items, and reactive aggression, containing 11 items. Subscale scores were calculated, in the range of 0 to 24 for proactive aggression and 0 to 22 for reactive aggression. The internal consistency of the instrument is good, with Cronbach’s alpha scores of 0.86 for the Proactive Aggression and 0.84 for the Reactive Aggression subscales, respectively [21] The Dutch version of the RPQ demonstrated good test–retest stability and adequate convergent and discriminant validity [30].

The Aggression Questionnaire—Short Form (AQ-SF) [31] is a short version of the Aggression Questionnaire [32]. It is a 12-item, self-report questionnaire that measures various subtraits of aggression. The Dutch version of the questionnaire (AVL-AV) was used [33]. The participants rated each item on a 5-point Likert scale, ranging from entirely disagree (1) to entirely agree (5). The questionnaire consists of four subscales: physical aggression, verbal aggression, anger, and hostility. The three item scores per subscale were summated to form a subscale score, with a maximum range of 3 to 15. Next, the four subscale scores were summated to achieve a total aggression score, in the range of 12 to 60. For secondary analyses, we dichotomized the total aggression scores, using the value at +1 standard deviation in our sample as a cut-off. The internal consistency coefficients of the subscales varied between 0.72 and 0.88, which indicates acceptable to good reliability. Adequate validity of the Dutch version of the AQ-SF was demonstrated by correlations with concurrent measurements [33].

As covariates, we measured age, gender, country of birth and nationality, marital status, offspring, educational level, and professional status. The therapist was requested, with written permission provided by the participant, to reconfirm the clinical *DSM–5* classification autism spectrum disorder and to provide information about comorbid diagnoses at the time of completing the questionnaire. Finally, we registered each participant as being treated in either outpatient or inpatient facilities, in which assisted living facilities with permanent supervision were listed as inpatient facilities.

### 2.4. Statistical Analysis

For all statistical analyses, we used SPSS version 25.0 [34]. For subgroup comparisons of sample characteristics, Student’s *t*-tests, Mann–Whitney *U* tests, or Pearson’s chi-squared tests were applied. To investigate the association of sensory processing and aggressive behavior, multiple linear regression models were used, including gender, age, educational level, and treatment setting as covariates. We added these specific covariates to all regression models to control for potential confounding effects. Previous research indicates that these variables are associated with at least one of the main variables: gender with aggressive behavior [35], age with sensory processing [10], and educational level (a proxy for IQ level) with aggressive behavior [36]. Treatment setting reflects the level of functioning and severity of symptoms. As we differentiated seven aggression-related outcome variables, we conducted an equal number of linear regression analyses with AASP raw quadrant scores and covariates as predictors. The more experimental AASP total score, the sensory deviation score, and the combination of neurological threshold and behavioral response were analyzed using additional linear regression analyses.

In line with our expectations, introducing the four raw quadrants scores induced multicollinearity. Table 2 shows Pearson’s correlation coefficients between the four AASP raw quadrant scores. In particular, sensory avoiding and sensory sensitivity were highly correlated.

The multicollinearity problem was expressed in high condition index values (>30), which also remained high after analyzing model versions with three out of four raw quadrant scores. To solve the multicollinearity problem, we applied forward selection as model building strategy and subsequently, if necessary, backward deletion. Forward selection was not needed in the analyses with the AASP total score and the sensory deviation score and with the neurological threshold and behavioral response scores as independent variables. Effect sizes of the predictor variables are expressed in Cohen’s $f^{2}$ and interpreted according to Cohen’s guidelines [37]. We analyzed dichotomized scores for total aggression and sensory processing using Fisher’s exact test. The missing data were limited to one missing data point on all of the main variables.

## 3. Results

### 3.1. Descriptive Statistics

The mean scores and standard deviations for the AASP total, sensory deviation, neurological threshold, behavioral response, and raw quadrant scores are presented in Table 3. Females on average had higher scores than males on the quadrants low registration, sensory avoiding, and sensory sensitivity. Females also had a higher AASP total score and sensory deviation score and lower scores on the neurological threshold and behavioral response. In comparison to inpatients, the outpatient group had higher mean scores on the quadrants sensory avoiding and sensory sensitivity, as well as the AASP total score and the sensory deviation score, and lower scores on the neurological threshold and behavioral response. We found no indication of statically significant differences between males and females on the various aggression scores. Participants treated in outpatient settings showed higher scores on total aggression, reactive aggression, and anger in comparison with inpatients.

**Figure 1:**
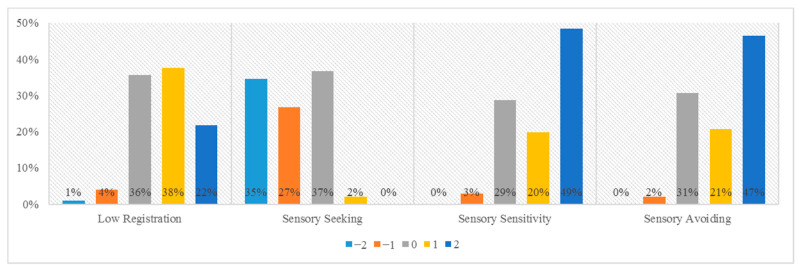
Percentage of AASP quadrant scores per distance from the AASP norm group. The labels −2 to 2 are used as equivalent for the AASP’s descriptions for norm group comparison, ranging from ‘much less than most people’ to ‘much more than most people’.

The mean sensory deviation score was 4.2, meaning that participants in our sample differed on average 4.2 standard deviations from the norm group on the total of the quadrant scores. In our sample, 97 out of 101 participants showed at least one standard deviation difference from the norm group on the quadrant scores. Half of the participants (51 out of 101) showed at least 5 standard deviations difference, and 4 participants had a maximum sensory deviation score of 8. Standardized quadrant scores are graphically presented in Figure 1. Scores on low registration were in majority 1 or 2 standard deviations higher than the norm group scores. In contrast, the majority of scores on sensory seeking were on minus 1 or minus 2 standard deviations in comparison to the norm group. The majority of scores on both sensory avoiding and sensory sensitivity were 1 or 2 standard deviations higher than the norm group scores.

**Figure 2:**
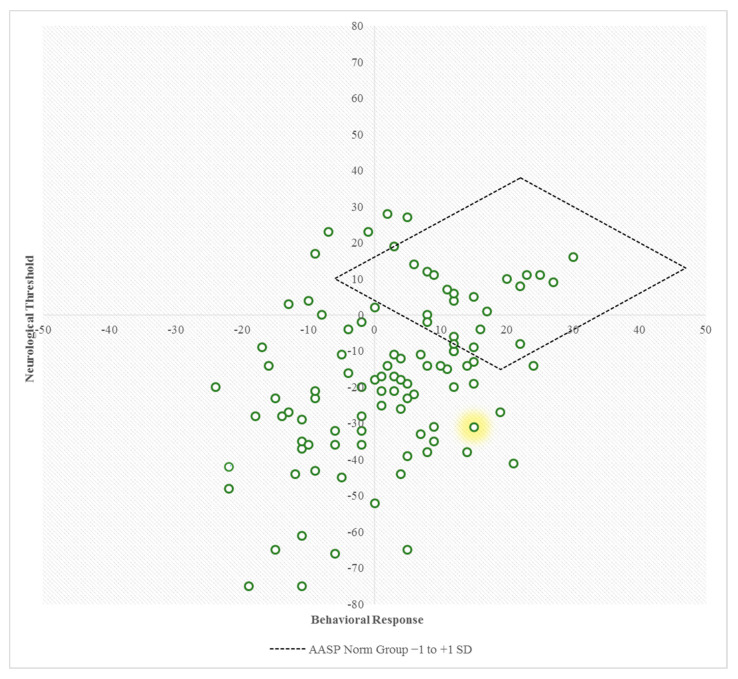
Scatterplot based on the calculated neurological threshold and behavioral response scores. The diamond shaped region represents the norm group’s neurological threshold scores and behavioral scores, as calculated with the norm group’s four quadrant score ranges between −1 and +1 SD. The highlighted data point represents patient Z from the example presented.

The scatterplot in Figure 2 plots each participant using the calculated neurological threshold and behavioral response scores. The majority of participants had a neurological threshold below zero—thus, at the lower threshold half. The points were fairly evenly distributed over the passive and active sides of the behavioral response axis. The variance in the neurological threshold was larger than the variance in behavioral response. The majority of the participants in our sample tended to have a lower neurological threshold and a more passive behavioral response than the norm group (represented in the diamond shaped region in Figure 2).

Example. Participant Z is a 25-year-old female with ASD, treated in a specialized inpatient facility for adults with ASD. Her AASP quadrant scores were 32 on low registration, 48 on sensory seeking, 56 on sensory sensitivity, and 55 on sensory avoiding. Norm group comparison demonstrated that her scores on low registration and sensory seeking are around the mean norm scores, but her scores on sensory sensitivity and sensory avoiding are much higher than the mean norm group scores. As a result, her sensory deviation score equals 4. The summation of her quadrant scores results in an AASP total score of 191. Using the formula (low registration + sensory seeking) − (sensory sensitivity + sensory avoiding), her neurological threshold is (32 + 48) − (56 + 55) = −31. Using the formula (sensory seeking + sensory avoiding) − (low registration + sensory sensitivity), her behavioral response score equals (48 + 55) − (32 + 56) = 15. The negative (low) neurological threshold score and positive (active) behavioral response score resulted in her representation in the predominantly sensory avoiding quadrant in Figure 2.

### 3.2. Regression Analysis

Results of the various multiple linear regression analyses are presented in Table 4. For the specific types of aggressive behavior, we found medium to large effects in models with reactive aggression and anger as dependent variables, and scores on sensory sensitivity and sensory seeking as independent variables. Adding low registration and sensory seeking to a model with proactive aggression as dependent variable resulted in a medium effect size. Medium effects were also observed in models with sensory sensitivity associated with verbal aggression and low registration associated with hostility. Sensory avoiding was deleted from all models after applying forward selection.

The AASP total score was positively associated with total aggression score and all specific types of aggressive behavior. Our other measure of general sensory processing difficulties, the sensory deviation score, was also positively associated with total aggression, as well as with verbal aggression, anger, and hostility. Whereas the behavioral response score was statistically significantly associated with total aggression, we found no indication for an association of the neurological threshold with total aggression. However, adding the interaction of neurological threshold with behavioral response scores in the model revealed statistical evidence for effect modification. This means that the behavioral response is associated with aggression in specific ranges of the neurological threshold. On the level of raw quadrant scores, both the sensory sensitivity score and the low registration score were positively associated with total aggression. We identified nineteen participants at the highest risk for demonstrating aggressive behavior (≥1 SD). All nineteen participants had elevated scores on sensory sensitivity (≥1 SD;N=69;FET p=0.001). Fifteen out of the nineteen participants with the highest scores on total aggression had elevated scores on low registration (≥1 SD;N=60;FET p=0.071).

## 4. Discussion

In this study, we found evidence for the association between sensory processing difficulties and aggressive behavior in adults with autism spectrum disorder. Individuals with more sensory processing difficulties showed higher levels of aggressive behavior. In-depth analysis revealed that adults with ASD with higher sensory sensitivity are more likely to show reactive aggression and anger, whereas those with difficulties concerning low registration of sensory input showed more proactive aggression. Adults with ASD who had increased levels of sensory seeking behavior showed both more proactive and reactive aggression, as well as more anger. We found evidence for an interaction between neurological threshold and behavioral response on total aggression and hostility. Finally, we found that the adults with ASD who had higher scores in comparison to the norm group in sensory sensitivity had the highest risk of aggressive behavior.

Our results confirmed findings from previous research with regard to the association of sensory processing difficulties and aggression in children [4,38] and in adults with ASD and comorbid intellectual disabilities [16]. Our study broadens these previous findings by suggesting that sensory processing difficulties are positively associated with more behavioral problems [39]. In previous research in children with ASD, sensory sensitivity was associated with externalizing behavior in typically developing children [40]. Aggression towards others in children with ASD was associated with low registration [38], whereas aggression was associated with sensory seeking behavior in low-functioning adults with ASD [16]. Our results add to this field of research by showing that reactive and proactive aggression differentially relate to different levels of sensitivity to sensory stimulation and responses to under- or overstimulation. This has clinical relevance as it helps to explain why aggressive behavior is displayed, allowing for an effective substitution by less disturbing coping mechanisms that address sensory issues.

The most robust associations were found between the AASP total score and aggression. This total score has been used in previous research [41] and overcomes the problem of collinearity between raw quadrant scores. In our sample, we found that, compared to the normal population, low registration, sensory sensitivity, and sensory avoiding in adults with ASD tend to be similar or higher, whereas sensory seeking tends to be similar or lower.

Although direct causal inferences cannot be drawn from a cross-sectional study, it is tempting to speculate about underlying mechanisms. Low registration is often described as being marked by missing sensory input and more passive self-regulation strategies. This passivity may induce less involvement in situations in which more externally directed forms of aggressive behavior could occur. However, adults with ASD with low registration may still feel easily overstimulated, by ruminating thoughts for example, which is reflected in higher scores on the internal state of hostility. Interestingly, the more externally directed forms of aggression, i.e., anger as well as proactive and reactive aggression, were all associated with sensory seeking behavior, suggesting that this sensory seeking behavior is to some extent conditional for these types of aggression, or tends to be involved in situations in which these types of aggression can occur. Concerning anger and reactive aggression, the combination of sensory seeking and sensory sensitivity suggests that hypersensitivity is relevant to induce these types of feelings and behavior. Hypersensitivity may function as a trigger for reactive aggression and feelings of anger. With regard to proactive aggression, our results suggest that it is the combination of sensory seeking with low registration—and perhaps associated hostility—that underlies overt aggression in adults with ASD.

Adults with ASD are known to have more sensory processing difficulties compared to the general population [14]. Our results confirm that individuals with ASD also present with significantly different scores in the quadrants as calculated with the AASP, thus showing differences in the more specific areas of sensory seeking, sensory sensitivity, sensory avoiding, and low registration. By graphical presentation of the calculated neurological threshold and behavioral response [29], we offered an innovative view on the sensory profiles in persons with ASD compared to the norm population, which shows that the majority shows a lower neurological threshold and more passive behavioral response.

Detailed measurement of aggressive behavior allowed us to demonstrate that different types of aggressive behavior are related to different aspects of sensory processing. Coarse meshed comparison with previous studies, without possible statistical inference, indicates that the various aggression-related scores in our sample are relatively similar to those in the general population [30,33]. We found no indication for differences between men and women with ASD. Although we would have expected higher levels of physical aggression in men and, perhaps, higher levels of anger or hostility in women [35], we did not find evidence for gender-specific vulnerabilities in persons with ASD. The confounding effects of gender were limited—despite demonstrated differences in sensory processing variables—although our analyses were corrected for gender. Our findings indicate gender-related differences in sensory processing, but no significant differences in the magnitude or direction of associations between sensory processing and aggressive behavior. Therefore, we have no indication that the generalizability of our findings to other (clinical) populations with more male persons with ASD would be limited.

Some limitations need to be discussed. First, selection bias could have been present due to differences in commitment to scientific research between therapists, but only when this commitment is related to patient characteristics. However, we found no clear indication that our study sample differed from our source population. Second, our source population at Dimence might not be fully representative of the broad population of adults with ASD, due to the effect of the institution’s specializations and expertise. Our source institution provides general mental health care but also accommodates teams specialized in mental health care for normal- to higher-functioning adults with ASD with complex comorbidity, such as forensic–psychiatric problems or addiction. In part, we analyzed the relevance of these factors as potential confounding variables or effect modifiers. In general, our results might be generalizable to the ASD populations of normal- to higher-functioning individuals, including women in both inpatient and outpatient settings. Third, we had no information available on current medication use, which is a possible confounding factor. There is some evidence to suggest that psychotropic medications, e.g., atypical antipsychotics such as risperidone, which is used to treat irritability and maladaptive behavior in children with ASD [42], may also impact aspects of sensory processing, either by heightening neurological thresholds or by influencing behavioral response. Finally, the potentially relevant factors ASD severity level and intelligence were not available in our dataset, which may have resulted in unmeasured confounding effects. We used available proxy measurements, respectively treatment setting and educational level.

In sum, clinical practice may benefit from applying detailed assessment of sensory processing problems when treating aggressive behavioral problems in adults with ASD. Therapists and patients may use the sensory profile as an alternative treatment target in case of unexplained or treatment-resistant aggressive behavior. Future research on the added value of the calculated neurological threshold and behavioral response, as well as the mapping of the individual on the model of sensory processing, may further inform clinical practice. Finally, we would recommend replication of our findings in broader populations of (forensic) psychiatric patients, e.g., patients with attention-deficit/hyperactivity disorder or learning disabilities, to further unravel underlying mechanisms in understanding the relation between sensory processing difficulties and aggression.

## 5. Conclusions

In our study, we demonstrated the association between sensory processing and aggressive behavior. Evidence was presented for this association on more detailed levels of both sensory processing and aggressive behavior. Additionally, we presented new methods for calculating and presenting the results of the Adolescent/Adult Sensory Profile. Clinical practice may benefit from applying detailed diagnostics on sensory processing difficulties when treating aggressive behavior in adults with ASD.

## Figures and Tables

**Table 1:** General sample characteristics for total sample and by sex and treatment setting.

Descriptive		Total N = 101	Males N = 53	Females ^A^ N = 48			Outpatient N = 71	Inpatient ^A^ N = 30		
Age (*M*, *SD*)		32.9 12.4	35.4 14.7	30.1 8.3	t=2.27 ^B^	*	34.5 12.8	29.1 10.4	t=2.02	*
Educational	Lower (%)	34.7	37.7	31.3	U=1156		23.9	60.0	U=684	**
level	Middle (%)	49.5	49.1	50.0			57.7	30.0		
	Higher (%)	15.8	13.2	18.8			18.3	10.0		
Paid work (%)		21.8	22.6	20.8	$x^{2}=0.05$		26.8	10.0	$x^{2}=3.48$	
Partner (%)		22.8	17.0	29.2	$x^{2}=2.13$		28.2	10.0	$x^{2}=3.96$	*
Children (%)		17.8	24.5	10.4	$x^{2}=3.43$		25.4	0.0	$x^{2}=9.26$	**
Comorbidity	Depressive/anxiety disorder (%)	36.6	35.8	37.5	$x^{2}=0.03$		39.4	30.0	$x^{2}=0.81$	
	ADHD (%)	13.9	15.1	12.5	$x^{2}=0.14$		14.1	13.3	$x^{2}=0.01$	
	Other (%)	30.7	30.2	31.3	$x^{2}=0.01$		29.6	33.3	$x^{2}=0.14$	

* p<0.05, ** p<0.01; ^A^ Test of sex and treatment setting: $x^{2}=0.97,\;p=0.33$. ^B^ *t* test with equal variances not assumed.

**Table 2:** Pearson’s correlation coefficients between AASP raw quadrant scores.

	Low Registration	Sensory Seeking	Sensory Sensitivity
Sensory Seeking	0.26 **		
Sensory Sensitivity	0.57 **	−0.07	
Sensory Avoiding	0.44 **	−0.29 **	0.81 **

** p<0.01.

**Table 3:** AASP, AQ-SF and RPQ mean scores (standard deviation) for total sample and by sex and treatment setting.

Variable	Total	Males	Females			Outpatient	Inpatient		
Low Registration	38.0 (8.4)	36.0 (8.1)	40.1 (8.3)	t=−2.50	*	39.0 (8.0)	35.5 (9.0)	t=1.95	
Sensory Seeking	39.1 (8.8)	40.1 (8.6)	38.1 (8.8)	t=1.16		39.1 (8.2)	39.1 (10.0)	t=0.01 ^A^	
Sensory Sensitivity	46.7 (11.3)	42.1 (10.6)	51.7 (9.9)	t=−4.69	**	49.0 (10.2)	41.0 (11.9)	t=3.43	**
Sensory Avoiding	47.9 (10.6)	45.0 (10.1)	51.2 (10.3)	t=−3.07	**	49.6 (10.2)	43.9 (10.5)	t=2.53	*
AASP Total Score	171.7 (27.2)	163.2 (27.1)	181.0 (24.2)	t=−3.49	**	176.8 (24.2)	159 (30.3)	t=3.03	**
Sensory Deviation Score	4.2 (2.2)	3.5 (2.0)	5.0 (2.1)	t=−3.56	**	4.5 (2.1)	3.5 (2.1)	t=2.26	*
Neurological Threshold	−17.5 (22.4)	−11.0 (20.3)	−24.7 (22.6)	t=3.21	**	−20.5 (21.9)	−10.4 (22.3)	t=−2.11	*
Behavioral Response	2.4 (12.1)	6.9 (10.6)	−2.5 (11.8)	t=4.24	**	0.7 (12.3)	6.5 (10.8)	t=−2.26	*
Total Aggression	28.0 (9.8)	27.5 (9.1)	28.5 (10.6)	t=−0.53		29.4 (9.9)	24.6 (8.7)	t=2.31	*
Reactive Aggression	8.5 (5.1)	7.7 (4.2)	9.4 (5.8)	t=−1.73 ^A^		9.4 (5.0)	6.5 (4.6)	t=2.73	**
Proactive Aggression	1.5 (1.9)	1.7 (1.8)	1.3 (1.9)	t=1.08		1.6 (1.9)	1.3 (1.8)	t=0.83	
Physical Aggression	5.7 (3.2)	5.8 (3.3)	5.7 (3.2)	t=0.19		6.0 (3.4)	5.2 (2.9)	t=1.08	
Verbal Aggression	6.2 (2.5)	6.0 (2.0)	6.3 (2.9)	t=−0.62 ^A^		6.3 (2.5)	5.9 (2.6)	t=0.61	
Anger	7.2 (3.7)	6.6 (3.6)	7.8 (3.8)	t=−1.64		8.1 (3.6)	5.2 (2.9)	t=3.86	**
Hostility	8.9 (3.6)	9.0 (3.4)	8.7 (3.8)	t=0.49		9.1 (3.7)	8.3 (3.3)	t=1.04	

* p<0.05, ** p<0.01; ^A^ *t*-test with equal variances not assumed.

**Table 4:** Multiple regression models with AASP raw quadrant, total, sensory deviation, neurological threshold and behavioral response scores and specific types of aggressive behavior.

	Total Aggression	Proactive	Reactive	Physical	Verbal	Anger	Hostility
Low Registration	b=0.301 * (0.049, 0.552)	b=0.047 * (0.001, 0.093)	^A^	b=0.106 ** (0.028, 0.185)	^A^	^A^	b=0.142 ** (0.058, 0.227)
Sensory Seeking	^A^	b=0.060 ** (0.017, 0.102)	b=0.179 ** (0.077, 0.280)	^A^	^A^	b=0.079 * (0.006, 0.152)	^A^
Sensory Sensitivity	b=0.272 * (0.055, 0.488)	^A^	b=0.172 ** (0.078, 0.266)	^A^	b=0.089 ** (0.038, 0.140)	b=0.129 ** (0.062, 0.197)	^A^
Sensory Avoiding	^A^	^A^	^A^	^A^	^A^	^A^	^A^
Effect Size ^B^	$f^{2}=0.25$	$f^{2}=0.19$	$f^{2}=0.27$	$f^{2}=0.08$	$f^{2}=0.13$	$f^{2}=0.20$	$f^{2}=0.12$
AASP Total Score	b=0.181 ** (0.109, 0.252)	b=0.019 * (0.004, 0.035)	b=0.082 ** (0.045, 0.119)	b=0.035 ** (0.009, 0.061)	b=0.040 ** (0.021, 0.059)	b=0.057 ** (0.031, 0.083)	b=0.048 ** (0.021, 0.076)
Effect Size ^B^	$f^{2}=0.27$	$f^{2}=0.07$	$f^{2}=0.21$	$f^{2}=0.07$	$f^{2}=0.18$	$f^{2}=0.20$	$f^{2}=0.13$
Sensory Deviation Score	b=1.611 ** (0.680, 2.542)	b=0.025 (−0.167, 0.217)	b=0.378 (−0.117, 0.873)	b=0.235 (−0.094, 0.565)	b=0.317 * (0.069, 0.565)	b=0.469 ** (0.133, 0.806)	b=0.589 ** (0.246, 0.932)
Effect Size ^B^	$f^{2}=0.12$	-	-	-	$f^{2}=0.07$	$f^{2}=0.08$	$f^{2}=0.12$
Neurological Threshold	b=−0.006 (−0.103, 0.090)	b=0.022 * (0.003, 0.040)	b=0.008 (−0.043, 0.058)	b=0.016 (−0.017, 0.049)	b=−0.016 (−0.041, 0.010)	b<0.001 (−0.035, 0.034)	b=−0.006 (−0.041, 0.029)
Behavioral Response	b=−0.202 * (−0.391, −0.013)	b=−0.013 (−0.049, 0.024)	b=−0.039 (−0.137, 0.059)	b=−0.049 (−0.114, 0.015)	b=−0.001 (−0.051, 0.049)	b=−0.062 (−0.129, 0.006)	b=−0.090 * (−0.158,−0.021)
Effect Size ^B^	-	-	-	-	-	-	$f^{2}=0.08$
Neurological Threshold x Behavioral Response	b=−0.010 ** (−0.017,−0.003)	b=−0.001 (−0.002, 0.001)	b=−0.003 (−0.007, 0.000)	b=−0.002 (−0.004, 0.001)	b=−0.001 (−0.003, 0.001)	b=−0.003 * (−0.005, <−0.001)	b=−0.004 ** (−0.007, −0.002)
Effect Size ^B^	$f^{2}=0.15$	-	-	-	-	$f^{2}=0.10$	$f^{2}=0.21$

* p<0.05, ** p<0.01. ^A^ Variable excluded from analysis after application of the forward selection method. ^B^ Effect size of $∆R^{2}$ after adding predictor variable(s) in the second block in comparison to the covariates in the first block. Only presented in case of statistically significant $∆R^{2}$.

## Data Availability

The data presented in this study are available on request from the corresponding author. The data are not publicly available due to privacy regulations.
